# Second law assessment of di methyl ether and its mixtures in domestic refrigeration system

**DOI:** 10.1038/s41598-023-27600-9

**Published:** 2023-01-06

**Authors:** A. Baskaran, N. Manikandan, N. Nagaprasad, Krishnaraj Ramaswamy

**Affiliations:** 1 Department of Mechanical Engineering, P.A. College of Engineering and Technology, Pollachi, 642002 Tamilnadu India; 2 Department of Mechanical Engineering, ULTRA College of Engineering and Technology, Madurai, 625104 Tamilnadu India; 3 Centre for Excellence-Indigenous Knowledge, Innovative Technology Transfer and Entrepreneurship, Dambi Dollo University, Dambi Dollo, Ethiopia; 4 Department of Mechanical Engineering, Dambi Dollo University, Dambi Dollo, Ethiopia

**Keywords:** Energy science and technology, Engineering, Materials science

## Abstract

Dimethyl ether (DME) and its blend of refrigerants (R429A, R435A, and R510A) are considered in this study's second law analysis as potential replacements for R134a. The performance of various refrigerants in a vapour compression refrigeration system is examined using the Design package CYCLE D. The software REFPROP 9.0 is used to extract all of the thermal and physical parameters of DME and its blend of refrigerants. The Second law performance parameters such as Efficiency Defects, Entropy generation and ExergyEfficiency are discussed. The refrigerants R429A and R510A are more energy efficient than R134a across a condensing temperature range of 30 to 55 °C at − 10 °C evaporation temperature. R134a was exceeded by R429A and R510A in terms of exergetic efficiency by 2.08 and 0.43%, respectively. In comparison to other losses in different components, the compressor's exergy loss is larger at 37–40% of the total exergy loss. By employing RE170 and its blends, the Vapour Compression Refrigeration System often performs better under the second law than R134a.

The result shows that the efficiency defects in the compressor are the largest, followed by the condenser and evaporator. Thus, the design improvement of a compressor is of at most importance to improve the system performance by lowering the overall irreversibility.

## Introduction

R134a is effectively used in domestic refrigerators (GWP 1430) as an alternative to CFC, which has high ODP and GWP^1,2^. The Kyoto Protocol from 1997 designated it as a greenhouse gas; thus, its production and use will end within the next decades. As a result, ecologically friendly refrigerants will take their place^3,4^. According to EU regulations, it is now essential to find a replacement refrigerant with a low GWP^5,6^. Table 1 lists the physical characteristics of the refrigerants under investigation. According to Nicholas Cox^7^, the absence of temperature glide and separation makes the Di methyl ether perform better than the hydrocarbon blend. Valentinapostol et al.^8^ make a comparative thermodynamic analysis using refrigerants R717, R12, R134A, R22, DME, and mix R404A, R407C in a refrigeration system. DME could be utilized as a refrigerant and a good replacement for R12 and R134a, according to the findings of this study.

**Table 1:** Properties of investigated refrigerants.

Refrigerant	R134a	RE170	R 429A	R 435A	R 510A
Composition (% mass f.)	–	–	RE170/R600a/R152a (60/30/10)	RE170/R 152a (80/20)	RE170/R 600a (88/12)
NBP(C)	− 26.1	− 24.8	− 26.0	26.1	− 25.2
GWP	1430	10	14	< 31	< 3
Molecular mass	102.3	46.07	50.76	49.04	47.24
Critical temperature	101.1	127.2	123.5	125.2	127.9
Critical pressure	4.06	5.34	4.86	5.39	5.33

The Di methyl ether (DME, C2H6O), according to B.M. Adamson^9^, possesses a number of desirable characteristics as a replacement for R134a. A few of these are enhanced heat transfer capabilities, favourable pressure/temperature stability with natural lubricants, relatively inexpensive, and prompt access. It's also very eco-friendly, and it's compatible with the majority of materials used in refrigeration systems.

Various researchers^10–31^ analyzed the thermal performance of VCR systems with DME and its blends. The result indicates that the investigated refrigerants are conformed as possible alternatives to R134a. Ki-Jung Park et al.^32^ investigated the domestic water purifier performance using R429A. The results indicate that the discharge temperature of the compressor and energy consumption is 13.40 C and 28.9% lower when compared with R134a. Choedaeseong et al.^33^ investigated the performance of R435A (a combination of DME and R152a) as a replacement for R134a in-home water purifiers. In comparison to HFC 134a, the electricity usage and release temperatures were respectively 12.7% and 3.7 °C lower. Through the use of R510A, Ki-Jung Park et al.^34^ examined the performance of household water purifiers. The result indicates that the discharge temperature of the compressor and energy consumption is 3.70 C and 22.3% lower when compared with R134a^35,36^. In this study, the second law performance of the system is investigated with the refrigerants RE170, R429A, R435Aand R510A as possible alternatives to R134a.

## Materials and methods

**Figure 1:**
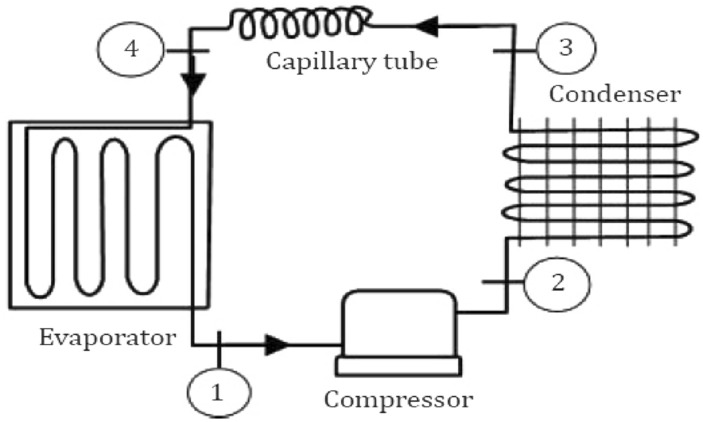
Block diagram of a refrigeration system.

**Figure 2:**
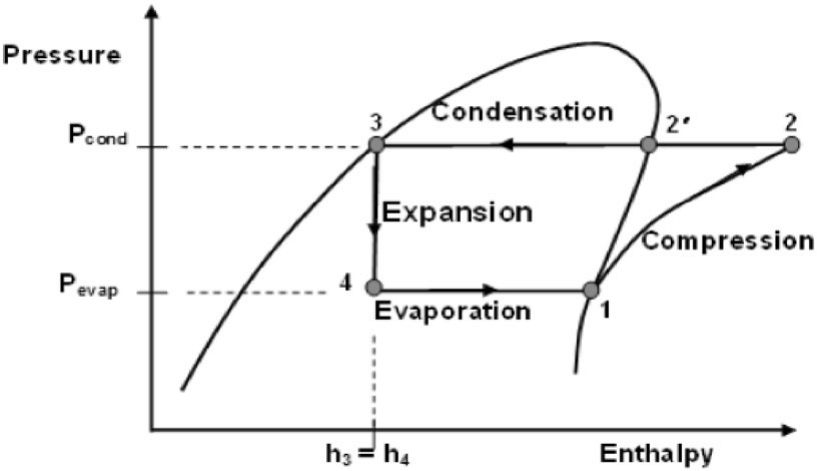
P–H diagram refrigeration system.

Figures 1 and 2 show the refrigeration system's block diagram and P–H diagram.

The details of the operating cycle are as follows.

- Isentropic compression (Stage 1–2).
- Condensation (Stage 2–3).
- Throttling (Stage 3–4).
- Evaporation (Stage 4–1).

The equations used for exergy analysis in different components:

- Exergy:
  1 $${\psi } = { }\left( {h - h_{0} } \right) - T_{0} \left( {s - s_{0} } \right)$$
- Evaporator:
- Abstraction of heat
  2 $$Q_{e} = m\left( {h_{1} - h_{4} } \right)$$
- Exergy losses,
  3 $$\begin{aligned} I_{ev} = & m\left( {{\uppsi }_{4} - {\uppsi }_{1} } \right) + Q_{e} \left( {1 - \frac{{T_{0} }}{{T_{ev} }}} \right) \\ = & m\left[ {\left( {h_{4} - h_{1} } \right) - T_{0} \left( {s_{4} - s_{1} } \right)} \right] + Q_{e} \left( {1 - \frac{{T_{0} }}{{T_{ev} }}} \right) \\ \end{aligned}$$
- Compressor:
- Exergy loss,
  4 $$\begin{aligned} I_{comp} = & m\left( {{\uppsi }_{1} - {\uppsi }_{2} } \right) + W_{e} \\ = & m\left[ {\left( {h_{1} - h_{2} } \right) - T_{0} \left( {s_{1} - s_{2} } \right)} \right] + W_{e} \\ \end{aligned}$$
- Condenser:
  5 $$Q_{cond} = m\left( {h_{2} - h_{3} } \right)$$
- Exergy loss,
  6 $$\begin{aligned} I_{cond} = & m\left( {{\uppsi }_{2} - {\uppsi }_{3} } \right) - Q_{cond} \left( {1 - \frac{{T_{0} }}{{T_{cond} }}} \right) \\ = & m\left[ {\left( {h_{2} - h_{3} } \right) - T_{0} \left( {s_{2} - s_{3} } \right)} \right] - Q_{cond} \left( {1 - \frac{{T_{0} }}{{T_{cond} }}} \right) \\ \end{aligned}$$
- Expansion Valve:
- Exergy loss,
  7 $$\begin{aligned} I_{exp} = & m\left( {{\uppsi }_{4} - {\uppsi }_{3} } \right) \\ = & m\left( {s_{4} - s_{3} } \right)\;\left[ {Thorttling,\;h_{4} = h_{3} } \right] \\ \end{aligned}$$
- Total exergy loss,
  8 $$I_{total} = I_{cond} + I_{exp} + I_{comp} + I_{ev}$$
- Efficiency defect:
- For Compressor,
  9 $${\updelta }_{comp} = \frac{{I_{comp} }}{{W_{el} }}$$
- Condenser,
  10 $${\updelta }_{cond} = \frac{{I_{cond} }}{{W_{el} }}$$
- Expansion valve,
  11 $${\updelta }_{exp} = \frac{{I_{exp} }}{{W_{el} }}$$
- Evaporator,
  12 $${\updelta }_{ev} = \frac{{I_{ev} }}{{W_{el} }}$$
- Exergy Efficiency,
  13 $${\upeta }_{x} = \frac{{{\uppsi }_{1} - {\uppsi }_{4} }}{{W_{el} }}$$
  14 $${\upeta }_{x} = 1 - \left( {{\updelta }_{comp} + {\updelta }_{cond} + {\updelta }_{exp} + {\updelta }_{ev} } \right)$$
  15 $${\text{S}}_{gen} = \frac{ I}{{T_{0} }}$$

For the application of R134a, Di methyl ether, and the following chosen mixtures, a theoretical study was carried out.

1. R510A is made up of 88% and 12%, a blend of RE170 and R600a.
2. R435A is made up of an 80% and 20% blend of RE170 and R152a
3. R 429A is made up of a 60%, 30%, and 10% mixture of RE170, R600a, and R152a.

The following conditions were taken into consideration when the behaviour of the vapour compression refrigeration system was examined using the CYCLE D 4.0 programme^36^.

- The isentropic and volumetric efficiency of the compressor = 0.75.
- Cooling capacity = 1.00 kW.
- The efficiency of the Electric motor = 0.75.
- Suction line heat exchanger efficiency = 0.80.
- Operating temperature of Evaporator =  − 50 °C to + 20 °C.
- Operating temperature of Condenser = 45 °C.
- Superheating temperature = 10 °C.

- Subcooling temperature = 5 °C.

**Figure 3:**
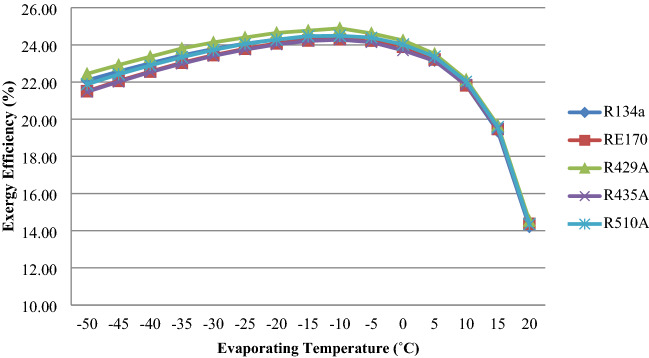
Exergy Efficiency as a Function of Evaporating Temperature.

**Figure 4:**
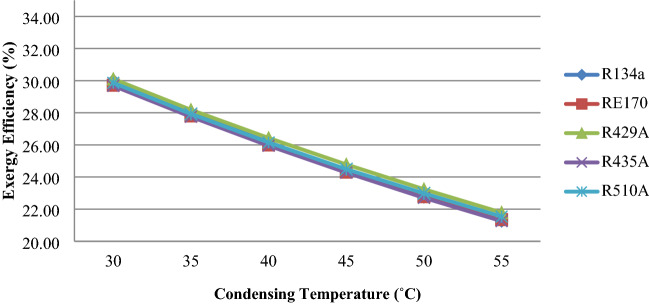
Exergy Efficiency and Condensing Temperature.

To obtain the enthalpy and entropy values required for the study, REFPROP 9.0 is employed^35^. This theoretical study examines the impact of energetic efficiency (Ex.eff) and efficiency flaws (Exd) in system components. The variation of Exergy Efficiency against the evaporating and condensing temperature is shown in Figs. 3 and 4, respectively. The variation of Efficiency defects (Exd) in system components are plotted in the figures from 5 to 6. The observations and Deviations of second law performance parameters in the VCR System are presented in Tables 2 and Table 3. Entropy generation in Various Components are presented in Table 4. Exergy losses in Various Components are listed in Table 5.

**Table 2:** Observations of performance parameters in VCR system.

S.NO	Performance parameters	R134a	RE170	R429A	R435A	R510A
1	Exergy efficiency	24.39	24.30	24.89	24.28	24.49
2	Compressor efficiency defect	0.3576	0.3503	0.3558	0.3502	0.3522
3	Condenser efficiency defect	0.2800	0.3017	0.2861	0.3012	0.2952
4	Expansion valve efficiency defect	0.0362	0.0260	0.0250	0.0270	0.0270
5	Evaporator efficiency defect	0.2396	0.2402	0.2448	0.2399	0.2416
6	Suction line heat exchanger efficiency defect	0.0156	0.0111	0.0099	0.0112	0.0121

Tcod = 45 °C, T evap =  − 10 °C, Temperature of superheat = 10 °C and Temperature of sub cool = 5 °C.

**Table 3:** Deviation of performance parameters (values are in percentage).

S.NO	Performance parameters	RE170	R429A	R435A	R510A
1	Exergy efficiency	− 0.33	2.08	− 0.45	0.43
2	Compressor efficiency defect	− 2.04	− 0.49	− 2.06	− 1.49
3	Condenser efficiency defect	7.76	2.18	7.59	5.43
4	Expansion valve efficiency defect	− 28.30	− 31.08	− 25.32	− 25.35
5	Evaporator efficiency defect	0.26	2.19	0.12	0.85
6	Suction line heat exchanger efficiency defect	− 28.86	− 36.48	− 28.46	− 22.15

**Table 4:** Entropy generation in various components (kW/K).

Components	R134a	RE170	R429A	R435A	R510A
Compressor	0.00014304	0.00014012	0.00014232	0.00014008	0.00014088
Condensor	0.000112	0.00012068	0.00011444	0.00012048	0.00011808
Expansion valve	0.00001448	0.0000104	0.00001	0.0000108	0.0000108
Evaporator	0.00009584	0.00009608	0.00009792	0.00009596	0.00009664
Suction line heat exchanger	0.00000624	0.00000444	0.00000396	0.00000448	0.00000484

**Table 5:** Exergy losses in various components (kW).

Components	R134a	RE170	R429A	R435A	R510A
Compressor	0.042912	0.042036	0.042696	0.042024	0.042264
Condensor	0.0336	0.036204	0.034332	0.036144	0.035424
Expansion valve	0.004344	0.00312	0.003	0.00324	0.00324
Evaporator	0.028752	0.028824	0.029376	0.028788	0.028992
Suction line heat exchanger	0.001872	0.001332	0.001188	0.001344	0.001452

## Result and discussions

### Effect of exergy efficiency in varying evaporating temperatures

Figure 3 indicates the effect of Exergetic efficiency (η*ex*) in changing temperatures for evaporation. When the temperature of the evaporator rises, the exergy efficiency furthermore increases up to the optimum evaporator temperature, and after that, it will decrease. The most Exergetic efficiency is achieved at optimum evaporator temperature. The difference in exergy efficiency is caused by two things. One is the exergy. The second issue is the work that must be done on the compressor. As the temperature of the evaporator rises, the compressor's work decreases. As a result, these two elements improve exergy efficiency until it reaches the optimum evaporator temperature, beyond which it drops. At lower evaporation temperatures, the refrigerant R429A has a greater energy efficiency than R134a. At increasing evaporation temperatures, the exergetic efficiency of all chosen refrigerants has improved. Over a wide range of evaporation temperatures, R429A has a higher Exergetic efficiency value than R134a. R429A has a 1.6–2.3% better exergetic efficiency than R134a.

### Exergy efficiency effect in varying condensing temperatures

The influence of condensation temperatures on exergetic efficiency (ex) is seen in Fig. 4. Exergetic efficiency decreases as condenser temperatures rise. At lower condensation temperatures, the refrigerant R429A has more energy efficiency than R134a. At higher condensation temperatures, the exergetic efficiency of all chosen refrigerants has improved. Over a wide range of condensing temperatures, R429A and R510A have higher exergetic efficiency than R134a. R429A has a 0.30–2.49% higher exergetic efficiency than R134a.

### Efficiency defect effects in different components of a system

#### Compressor efficiency defect

**Figure 5:**
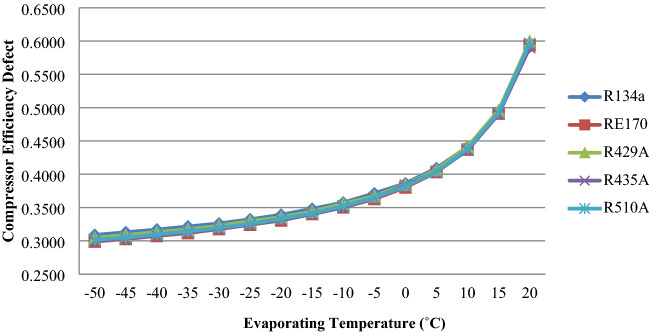
Compressor efficiency defect as a function of evaporator temperature.

With various evaporator temperatures, Fig. 5 depicts the effect of an efficiency flaw in the compressor for R510A, RE170, R429A, R435A, and R134a. The compressor efficiency defect grows as the temperature in the evaporator rises, as shown in the graph. The results show that R510A, R435A, R429A, and RE170 have less compressor efficiency defects than R134a.

### Condenser efficiency defect

**Figure 6:**
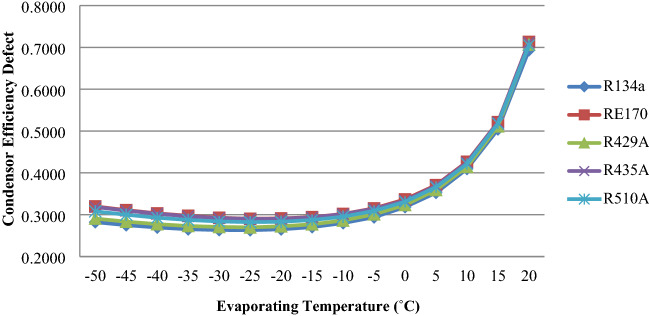
Condensor efficiency defect as a function of evaporator temperature.

Figure 6 indicates the effect of efficiency defect in the condenser for R510A, RE170, R429A, R435A and R134a with varying evaporator temperature. The figure reveals that the condenser efficiency defect reduces with rise in evaporator temperature up to − 25 °C and then increases. The result indicates that the condenser efficiency defects for investigated refrigerants are more than that of R134a.

### Expansion valve efficiency defect

**Figure 7:**
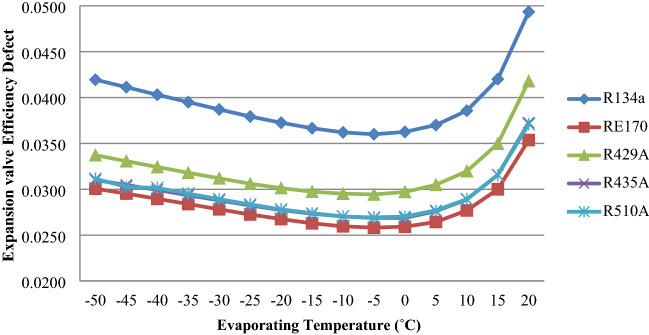
Expansion valve efficiency defect as a function of evaporator temperature.

For RE170, R429A, R435A, R510A, and R134a, Fig. 7 depicts the effect of an expansion valve efficiency failure as evaporator temperature varies. The expansion valve efficiency defect reduces with increasing evaporator temperature up to − 10 °C and thereafter increases, as seen in the graph. RE170, R429A, R435A, and R510A have lower expansion valve efficiency defects than R134a, according to the results^37–39^.

### Evaporator efficiency defect

**Figure 8:**
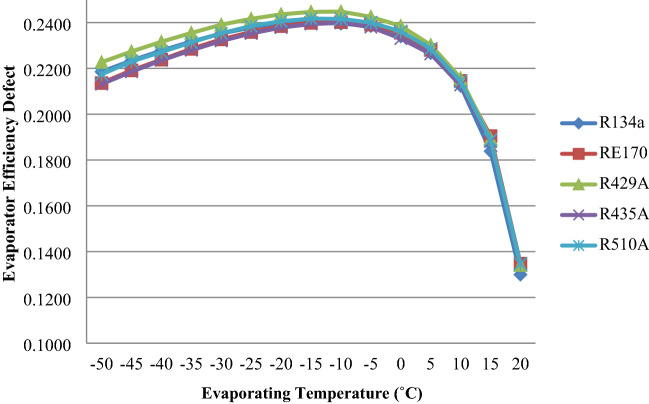
Evaporator efficiency defect as a function of evaporator temperature.

For RE170, R429A, R435A, R510A, and R134a, Fig. 8 depicts the effect of an evaporator efficiency fault as a function of evaporator temperature. The evaporator efficiency defect increases when the temperature in the evaporator rises to − 15 °C and then declines, as seen in the graph. The evaporator efficiency fault for R510A, R435A, R429A, and RE170 is higher than R134a, according to the results^40,41^.

### Suction line heat exchanger efficiency defect

**Figure 9:**
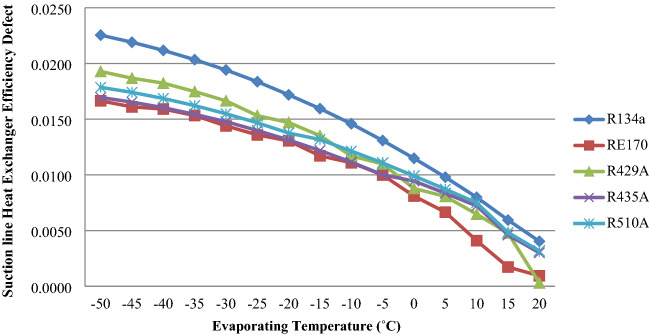
Heat exchanger efficiency defect as a function of evaporator temperature.

The effect of a suction line heat exchanger efficiency fault on evaporator temperature for RE170, R429A, R435A, R510A, and R134a is shown in Fig. 9. When the temperature in the evaporator rises, the efficiency defect in the suction line heat exchanger reduces, as shown in the graph. The results show that RE170, R429A, R435A, and R510A have lower suction line heat exchanger efficiency defects than R134a.The heat exchanger (suction line–capillary tube) achieved a good enhancement in COP and effectiveness compared with the reference capillary tube (without suction line) due to the increase in the sub-cooling zone^42–44^.

## Conclusions

The performance of the VCR system under the second law is investigated for the refrigerants R510A, R435A, R429A, and RE170. On the performance parameter, the impacts of evaporating temperature and condensing temperature are shown.

The observations in this analysis are given as follows.

- The refrigerants R429A and R510A are more energy efficient than R134a across a condensing temperature range of 30 to 55 °C at − 10 °C evaporation temperature.R429A and R510A had better exergetic efficiency than R134a by 0.31–2.46% and 0.37–1.29%, respectively.
- In compressor, the loss of exergy is 37–40% of the total exergy loss, which is higher than the other losses in various components.
- Mostly efficiency defect with R429A and R510A in the system is systematically better than R134a.
- The highest efficiency defects were obtained using selected refrigerants in Compressor, condenser and evaporator.
- By employing RE170 and its blends, the Vapour Compression Refrigeration System generally performs better under the second law than R134a.

## Data Availability

The datasets used and analysed during the current study available from the corresponding author on reasonable request.

## Abbreviations

DME: Di methyl ether
GWP: Global warming potential
ODP: Ozone layer depletion
NBP: Normal boiling point
HFC: Hydro fluro carbon
VCR: Vapour compression refrigeration
Ψ: Exergy (kW)
h: Enthalpy (kJ/kg)
T: Temperature (°C)
S: Entropy (kJ/kg.K)
m: Mass flow rate (kg/sec)
I_ev_: Irreversibility in evaporator (kW)
Q_e_: Refrigeration effect (kW)
T_ev_: Evaporator temperature (°C)
W_e_: Electrical power (kW)
Q_cond_: Heat dissipated in condensor (kW)
I_cond_: Irreversibility in condensor (kW)
T_cond_: Condensor temperature (°C)
I_total_: Total irreversibility (°C)
I_exp_: Irreversibility in expansion valve (kW)
I_com_: Irreversibility in compressor (kW)
δ_exp_: Efficiency defect in expansion valve
δ_com_: Efficiency defect in compressor
δ_con_: Efficiency defect in condensor
δ_ev_: Efficiency defect in evaporator
η_x_: Exergy efficiency
S gen: Entropy generation (kW/K)
